# High serum Antimullerian hormone levels are associated with lower live birth rates in women with polycystic ovarian syndrome undergoing assisted reproductive technology

**DOI:** 10.1186/s12958-020-00581-4

**Published:** 2020-03-10

**Authors:** Reshef Tal, Charles M. Seifer, Moisey Khanimov, David B. Seifer, Oded Tal

**Affiliations:** 1 Division of Reproductive Endocrinology & Infertility, Department of Obstetrics, Gynecology and Reproductive Sciences, Yale School of Medicine, 333 Cedar Street, New Haven, CT 06510 USA; 2 USF Health Morsani College of Medicine, Tampa, FL USA; 3 Genesis Fertility & Reproductive Medicine, Maimonides Medical Center, Brooklyn, NY USA; 4 School of Business and Hospitality, Conestoga College, Kitchener, Ontario Canada

## Abstract

**Introduction:**

Antimullerian hormone (AMH) strongly correlates with ovarian reserve and response to controlled ovarian stimulation. Emerging data suggests that serum AMH level may also predict ART outcomes. However, AMH is characteristically elevated in PCOS women and it is unknown whether it may predict live birth outcomes in this population.

**Methods:**

This was a retrospective cohort study of 184 PCOS women (Rotterdam criteria) who underwent their first fresh IVF/ICSI cycle. Women were divided into 3 groups according to the <25th (low), 25 to 75th (average), or > 75th (high) percentile of serum AMH concentration. Cycle stimulation parameters and reproductive outcomes were compared between groups.

**Results:**

Women in the low serum AMH group were older than those in the average or high AMH (*p* < 0.05), and required greater gonadotropin dose for stimulation compared to the high AMH group (p < 0.05). Women with high AMH had greater testosterone level compared to women in the low or average AMH groups. No differences were noted between groups in terms of maximal E2, oocytes retrieved and fertilization rate. However, low serum AMH women had significantly greater live birth rates (*p* < 0.05) and showed a trend towards greater clinical pregnancy rates compared to women in the average and high AMH groups (*p* = 0.09). The significant association of AMH with live birth rate remained after adjusting for age, BMI, day of transfer and number of embryos transferred.

**Conclusions:**

In PCOS women, elevated AMH concentrations are associated with hyperandrogenism and lower live birth rates.

## Introduction

Anti-Müllerian hormone (AMH), or Müllerian inhibiting substance (MIS), is a member of the transforming growth factor beta (TGF-β) superfamily. It is an established indicator of ovarian reserve and predictor of ovarian response in assisted reproductive technologies (ART) [1, 2]. AMH plays a role in folliculogenesis by inhibiting primordial follicular recruitment and FSH-dependent growth and selection of antral follicles [3]. AMH is produced exclusively in the granulosa cells of ovarian preantral and small antral follicles. In women with polycystic ovarian syndrome (PCOS) serum AMH is elevated, around 2 to 4-fold higher than normal, which is thought to be implicated in the pathogenesis of PCOS.

Polycystic ovarian syndrome affects 5–8% of reproductive age women. Diagnostic hallmarks of PCOS include oligo or anovulation, hyperandrogenism, and antral follicular excess on ultrasound [4]. Not only is follicular excess contributing to elevated AMH, but granulosa cells in women with PCOS have also been shown to overproduce AMH [5]. AMH levels are associated with severity of PCOS and correlate with all three diagnostic hallmarks of the disease, making it potentially useful as a diagnostic criterion for PCOS [6–12]. Based on the evidence for the association of AMH with PCOS pathophysiology, it has been investigated whether serum AMH may be related to assisted reproductive outcomes in women with PCOS.

There are many studies that have examined the link between serum AMH levels and IVF/ICSI outcomes such as implantation, clinical pregnancy, and live birth rate. AMH appears to be associated with IVF/ICSI outcomes but is overall a poor predictor of these outcomes [13–15]. However, the results of various studies are inconsistent, and many of these did not include women with PCOS. In studies that have looked at these relationships in PCOS women, the results are conflicting. Although some studies have suggested a positive relationship [11, 16], some others have suggested an inverse relationship [17] between AMH concentration and pregnancy rates. Other studies have gone further to look at these associations based on PCOS phenotypes [18–20], but no consensus has been reached. AMH is characteristically elevated in PCOS women and it is unknown whether it may predict ART outcomes in this population. Therefore, our goal was to determine whether serum AMH level is associated with ART cycle characteristics, pregnancy rates and live birth outcomes in women with PCOS.

## Materials and methods

### Study participants

This was a retrospective cohort study of eligible consecutive women which included 184 PCOS patients who underwent their first fresh IVF/ICSI cycle in our clinic between April 2009 and April 2014. Frozen embryo transfers were excluded. Women were stratified into 3 groups according to the <25th (low), 25 to 75th (average), or > 75th (high) percentile of serum AMH concentration. The women’s demographics, cycle characteristics and clinical and laboratory data were extracted from electronic medical records and included the following parameters: age, body mass index (BMI) (kg/m^2^), serum follicle-stimulating hormone (FSH), luteinizing hormone (LH), AMH, total testosterone, dehydroepiandrosterone sulfate (DHEAS), 17-OH-progesterone, thyroid-stimulating hormone (TSH), and prolactin. The diagnosis of PCOS was made when at least 2 of the following 3 criteria existed, as proposed by the Rotterdam Consensus Meeting [4]: oligomenorrhea or amenorrhea, clinical hyperandrogenism and/or hyperandrogenemia, and polycystic ovaries. PCOS diagnosis was established after exclusion of other endocrine conditions including hyperprolactinemia, thyroid disease, Cushing’s syndrome, congenital adrenal hyperplasia, or androgen-secreting tumors. This study was approved by our institutional review board.

Eligible patients were PCOS women between 18 and 40 years old and underwent their first fresh IVF/ICSI treatment cycle. Patients enrolled in an oocyte donation or gestational surrogacy program were excluded, as well as patients undergoing preimplantation genetic testing.

### Ovarian stimulation and fresh embryo transfer

Ovarian stimulation was performed using a combination of recombinant FSH (Follistim, Merck or Gonal-f, Serono) and human menopausal gonadotropin (Menopur, Ferring). The stimulation protocol included either pituitary downregulation via GnRH agonist in a long protocol or a GnRH antagonist to prevent premature ovulation. Dosage was determined by performing follicular tracking using ultrasound and blood sampling for estradiol levels every 1–3 days. When at least 6 follicles with a diameter of 16 mm were detected, either 5000 or 10,000 IU human chorionic gonadotropin (hCG) was administered as final oocyte maturation, depending on the estimated risk for hyperstimulation. 35 h following hCG administration, oocytes were retrieved under transvaginal sonographic guided needle puncture. In patients who were deemed to be at high risk for ovarian hyperstimulation syndrome (OHSS), all embryos were cryopreserved. Decision to extend culture of embryos day 5 (vs. day 3) was made at the physicians’ discretion according to the number of embryos of good morphological quality on day 3. Vaginal micronized progesterone was administered for luteal support until 10 weeks of pregnancy.

### AMH assay

Random serum AMH levels for each woman, unrelated to the day of the menstrual cycle, were measured by enzyme-linked immunosorbent assay (ELISA) at Reprosource Inc. (Woburn, MA) as previously described [21].

### Main outcome measures

The live birth rate (LBR) per fresh embryo transfer was the primary outcome parameter in this study, defined as the number of live birth events (> 24 weeks gestation) divided by the number of embryo transfer procedures. Secondary outcome measures were implantation rate and clinical pregnancy rate per embryo transfer. The implantation rate was calculated as the number of gestational sacs divided by the number of embryos transferred. Clinical pregnancy was defined as the presence of an intrauterine gestational sac on ultrasound performed at > 6 weeks after embryo transfer. The clinical pregnancy rate was calculated as the number of clinical pregnancies divided by the number of embryo transfer procedures.

### Statistical analysis

The subjects were stratified into three groups according to the <25th (*n* = 46), 25 to 75th (*n* = 92), or > 75th (*n* = 46) percentile of serum AMH concentration. Continuous data are expressed as mean ± standard deviation. Categorical data are presented by the number of cases and corresponding percentage. Mean differences between 2 groups were compared using Student t test or Mann-Whitney test, as appropriate. Baseline and cycle stimulation parameters as well as reproductive outcomes were compared between groups using Kruskal-Wallis multiple comparison or chi-square tests. A receiver operating characteristic (ROC) curve was generated to investigate the predictive value of AMH level for live birth rate. The sensitivity and specificity were calculated for the optimal AMH cutoff level determined by ROC curve analysis. Univariate analyses of the effect of various variables on live birth were performed using chi square tests for candidate variables that may interact with AMH. In addition, the odds ratio (OR) for live birth were adjusted for age, BMI, number of embryos transferred and day of transfer using the Cochran-Mantel-Haenszel test. The likelihood of live birth was described as an odds ratio with standard 95% confidence interval (CI). All significance tests were two-tailed and *P*-value < 0.05 were considered to be statistically significant. SigmaPlot (Systat Software Inc., Chicago, IL) and MedCalc software (Mariakerke, Belgium) were used for statistical analysis.

## Results

The main baseline characteristics are listed in Table 1, and cycle characteristics are listed in Table 2. AMH levels decreased with increasing age of women in our cohort, with women in the low AMH group being significantly older compared to the average and high AMH groups. While the literature suggests an inverse relationship between BMI and AMH [22], there was little variation in BMI between the different AMH level groups in this study. In agreement with previous literature, a direct relationship was found between testosterone and AMH levels [11, 17] with a significant increase in the high AMH group compared to the average and low AMH groups. Total testosterone was 35.3 ± 10.2, 39.2 ± 12.8, and 58.0 ± 18.7 ng/dl in the <25th, 25 to 75th, and > 75th percentile, respectively. There was an inverse relationship between AMH levels and gonadotropin dose, which was significant in the <25th percentile group (3316 ± 1288 IU) compared to the 25 to 75th percentile (2587 ± 1071 IU) and > 75th percentile (2252 ± 842 IU) groups. E2 levels measured on the day of hCG administration varied among the groups with little evidence for a trend.

**Table 1:** Baseline characteristics

Variable		Serum AMH (ng/ml)				
	PCOS Low, < 3.32 *n* = 46	PCOS Average, 3.32–8.27 *n* = 92	PCOS High, > 8.27 *n* = 46	*p*-value Low vs. High	p-value Low vs. Average	p-value Average vs. High
AMH (ng/ml)	2.1 ± 0.8	5.2 ± 1.3	12.8 ± 4.3	< 0.0001	< 0.001	< 0.0001
Age (yr)	33.9 ± 4.0	31.1 ± 3.5	30.6 ± 3.9	< 0.01	< 0.01	NS
BMI (Kg/m^2^)	26.5 ± 5.7	26.6 ± 5.8	26.9 ± 5.0	NS	NS	NS
Total Testosterone (ng/dl)	35.3 ± 10.2	39.2 ± 12.8	58.0 ± 18.7	< 0.05	NS	< 0.05
TSH (mU/L)	1.5 ± 0.7	1.9 ± 0.9	1.4 ± 0.8	NS	NS	NS
Prolactin (ng/mL)	12.6 ± 6.3	12.1 ± 10.4	10.2 ± 6.1	NS	NS	NS
Infertility etiology, n (%)				NS	NS	NS
Male factor	27 (58.6)	48 (52)	22 (47.8)			
Tubal factor	5 (10.9)	6 (6.5)	5 (10.9)			
Ovulatory	11 (23.9)	34 (36.9)	17 (37.0)			
Unexplained/other	3 (6.5)	4 (4.3)	2 (4.3)			

AMH, antimullerian hormone; BMI, body mass index; TSH, thyroid stimulating hormone

**Table 2:** Cycle characteristics and ART outcomes

Variable		Serum AMH (ng/ml)				
	Low, < 3.32 n = 46	Average, 3.32–8.27 n = 92	High, > 8.27 n = 46	*p*-value Low vs. High	p-value Low vs. Average	p-value Average vs. High
GnRH Antagonist (%)	81.2	72.3	68.8	NS	NS	NS
GnRH agonist (%)	18.8	27.7	31.2	NS	NS	NS
Total gonadotropin dose (IU)	3316 ± 1288	2587 ± 1071	2252 ± 842	< 0.01	< 0.05	NS
E2 (pg/ml) on day of hCG	2656 ± 964	2521 ± 880	2806 ± 1087	NS	NS	NS
Number of oocytes retrieved	11.8 ± 6.3	13.4 ± 6.2	14.6 ± 8.0	NS	NS	NS
Number of fertilizations	8.8 ± 4.9	8.6 ± 4.1	9.5 ± 5.6	NS	NS	NS
Number of embryos cryopreserved	1.8 ± 2.7	2.8 ± 2.6	3.2 ± 2.8	NS	NS	NS
Day 5 transfer (%)	78.1	70.8	71.1	NS	NS	NS
Number of embryos transferred	2.2 ± 0.7	1.9 ± 0.7	1.9 ± 0.8	NS	NS	NS
Fertilization rate (%)	70.4	64.2	66.5	NS	NS	NS
Implantation rate (%)	47.1	40.6	35.3	NS	NS	NS
Clinical pregnancy rate (%)	69.6	53.2	52.2	NS	NS	NS
Live birth rate (%)	65.2	46.7	43.5	< 0.05	< 0.05	NS
Multiple pregnancy rate (%)	26.0	21.7	13.0	NS	NS	NS
OHSS (%)	8.7	4.3	8.7	NS	NS	NS

GnRH, gonadotropin releasing hormone; OHSS, ovarian hyperstimulation syndrome; hCG, human chorionic gonadotropin; E2, estradiol;

From the low to high AMH ranges, the average number of oocytes retrieved increased from 12.6 ± 6.3, 13.4 ± 6.2, to 14.2 ± 8.0, respectively. The number of fertilizations was highest in the high AMH range as well at 9.5 ± 5.6. Approximately two embryos on average were transferred for each group per procedure.

**Fig. 1:**
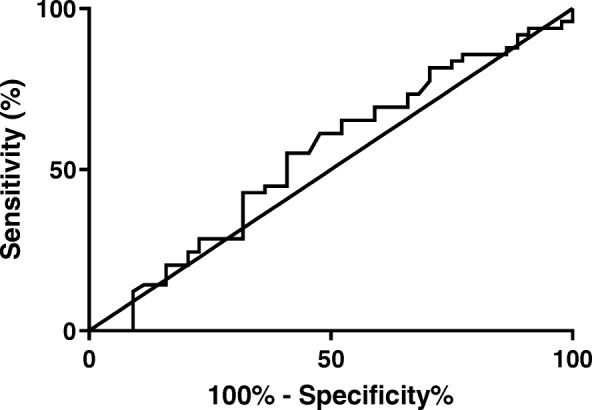
Receiver-operating characteristic (ROC) curve analysis for AMH as a predictor of live birth

In this study, there was significant increase in live birth rate per embryo transfer (LBR) in PCOS women in the low AMH group (65.2%) compared to the average AMH (46.7%) and high AMH (43.5%) groups. The odds ratio (OR) for LBR in the low AMH group compared to the other groups (average and high) was 2.29 (*p* = 0.017) and remained significant after adjusting for age, BMI, day of transfer and number of embryos transferred (Table 3). Receiver-operating characteristic (ROC) curve analysis for AMH as a predictor of live birth revealed an area under the curve (AUC) of 0.543 (0.424–0.661, 95% confidence interval) (Fig. 1) indicating overall poor predictability for live birth. Although not statistically significant, the same inverse trend with AMH concentration was found for clinical pregnancy rate (CPR) (69.6, 53.2, 52.2%), implantation rate (IR) (47.1, 40.6, 35.3%), and multiple pregnancy rate (26.0, 21.7, 13.0%). Fertilization rate was highest in the low AMH range (70.4%), but there was not a uniform inverse relationship with this outcome.

**Table 3:** Univariate analysis with odds ratios for live birth

Parameter	OR of live birth	95% CI	p-value
AMH level (low vs. average or high)	2.29	1.15–4.6	0.017
^a^Number of embryos transferred (1 vs. 2 or more)	1.74	0.88–3.82	0.17
^b^Age (21–31 vs. 32–40)	0.74	0.41–1.32	0.30
^c^Day of transfer (3 vs. 5)	0.74	0.37–1.47	0.38
^d^BMI (17–26 vs. 27–39)	1.14	0.64–2.04	0.66

^a^OR of low AMH for live birth remained significant after adjusting for number of embryos transferred (OR 2.29, *p*-value 0.021)

bOR of low AMH for live birth remained significant after adjusting for BMI (OR 2.25, p-value 0.015)

^c^OR of low AMH for live birth remained significant after adjusting for age (OR 2.23, p-value 0.038)

^d^OR of low AMH for live birth remained significant after adjusting for day of transfer (OR 2.29, p-value 0.016)

## Discussion

This study is the first to describe an association between serum AMH levels and live birth outcomes in PCOS women. Live birth outcome is the ultimate clinically important measure in ART treatment and the results of this study indicate that PCOS women with higher serum AMH levels have poorer IVF outcomes compared to PCOS women with low/normal AMH levels.

In the present study, women with low serum AMH levels were older than those with average and high serum AMH levels. This relationship agrees with previous studies, as serum AMH levels begin declining around 25 years of age to undetectable levels at menopause [23]. Total gonadotropin dose was significantly higher in women in the low serum AMH group than those in the average and high serum AMH groups as well. FSH stimulates the growth and development of follicles in early folliculogenesis. The exogenous dose was dependent on intermittent daily measures of estradiol and ultrasound analysis of follicular growth. In the low serum AMH group, the increased gonadotropin dose compensated for lesser follicular growth by increasing the number of follicles that reach maturity. Although the number of oocytes retrieved increased slightly from low to high serum AMH groups, the success of this approach was reflected in the relatively similar number of oocytes retrieved across all women despite the broad range of serum AMH levels. This finding is supported by previous studies, showing that serum AMH levels reflect the ovarian response to stimulation [1, 24]. The fertilization rate was not significantly different between AMH groups, although the total number of fertilizations was also highest in the high serum AMH group, which could be attributed to greater number of oocytes retrieved.

In our cohort of women with PCOS undergoing assisted reproduction, LBR was significantly higher in the low serum AMH group (< 3.32 ng/ml, 65.2%) than those with average (3.32–8.27 ng/ml, 46.7%) or high serum AMH levels (> 8.27 ng/ml, 43.5%). Similar trends were noted in implantation, CPR, and multiple pregnancy rate, which each showing an inverse relationship with serum AMH levels, albeit non-significant. To our knowledge, this study is unique in that it demonstrates that higher serum AMH levels are associated with lower assisted reproduction LBR in women with PCOS. There have been several investigations into the association between AMH and ART outcomes in women with PCOS, but they did not include live birth rate as an outcome [11, 16, 17]. These studies reported conflicting results, some demonstrating a positive relationship between serum AMH levels and CPR [11, 16], while others having found an inverse relationship between serum AMH levels and CPR [17]. The reason for this discrepancy is unclear. Sample size may account for the variation in outcomes. Notably, in the study by Kaya et al. [16] there were significant age differences between the three AMH strata: Women in the high AMH (>75th percentile), average AMH (25th–75th percentile) and low AMH (<25th percentile) groups had mean age of 25.1, 29.2 and 34.1, respectively. This was in contrast to the study by Xi et al. [17] as well as the current study in which all three AMH groups were of more comparable age, which may explain some of the observed differences. Another possible explanation is the presence of multiple PCOS phenotypes and variable AMH concentration categorizations across studies. A 2015 meta-analysis of women with and without PCOS concluded that AMH was a weak predictor of assisted reproduction outcomes in non-PCOS women but not in women with PCOS [15]. There are a few recent studies that more directly examined the connections between PCOS phenotypes, AMH levels and associated ART outcomes. In one study, AMH was found to have a significant positive correlation with CPR and LBR only in one out of four designated PCOS phenotypes but not in the general PCOS population comprised of all 4 phenotypes [20]. Another study which focused on whether PCOS phenotypes were associated with cumulative LBR concluded that hyperandrogenic phenotypes confer significantly lower cumulative LBR [19]. Additional studies which focus on categorizing these phenotypes may help elucidate the exact associations between AMH concentrations and ART outcomes in women with PCOS.

There are a few possible explanations for the correlation of high AMH and poor IVF outcomes in PCOS women. AMH regulates folliculogenesis in the ovaries [25], and it is elevated as a hallmark of PCOS and correlates with the severity of PCOS manifestations [11]. The 2 to 4-fold increase in serum AMH levels reflects the follicular arrest and higher preantral and small antral follicle count. In addition, granulosa cells within each follicle have been shown to overproduce AMH up to 75-fold the normal amount [5, 26]. Elevated AMH may cause abnormal folliculogenesis through two different mechanisms [27]. AMH inhibits FSH-dependent selection of a dominant follicle, leading to decreased granulosa cell FSH sensitivity and potential follicular arrest [28]. In addition, prior studies showed a positive correlation between AMH levels and degree of hyperandrogenemia [7, 10, 11, 29]. AMH inhibition of granulosa cell FSH-induced aromatase expression can lead to an accumulation of intraovarian androgens [30, 31]. Moreover, androgens play a role in stimulating early (FSH independent) stages of follicular growth [32, 33] and may therefore contribute to increased AMH production in the ovary. These two mechanisms may contribute to PCOS phenotypes of hyperandrogenism and anovulation. In our study, there was a significant increase in total testosterone levels (58.0 ± 18.7 ng/dl) in the high serum AMH group when compared to total testosterone levels in the average (39.2 ± 12.8 ng/dl) and low (35.3 ± 10.2 ng/dl) serum AMH groups. Consistent with these observations, in the studies by Ramezanali et al. [20] and De Vos et al. [19] it was shown that hyperandrogenic phenotypes in PCOS women were associated with poorer CPR and LBR. Furthermore, there is previous research that suggests androgen excess is associated with poor oocyte quality, leading to lower fertilizations rates [34].

Not only have androgen levels been shown to directly correlate with elevated serum AMH, but studies have also shown LH to directly correlate as well. LH stimulates AMH production in granulosa cells in women with PCOS [5], which may contribute to AMH overexpression and AMH induced follicular arrest. Another proposed mechanism involves LH induced suppression of FSH, causing poor oocyte and embryo quality [17, 35], leading to low implantation and clinical pregnancy rates associated with high AMH levels.

PCOS has been shown to have a detrimental effect on endometrial homeostasis and receptivity [36]. Moreover, women with high serum AMH are at increased risk for developing ovarian hyperstimulation syndrome, which can abnormally affect endometrial receptivity, thereby affecting IR and LBR. There is additional evidence that suggests certain angiogenic factors, like VEGF and TGF-β, are dysregulated in PCOS [37], which could further affect the endometrium. We speculate that elevated levels of AMH seen in PCOS leads to aberrant folliculogenesis as well as possibly contributing to altered endometrial receptivity and abnormal placentation. There is evidence that placenta and endometrial cells express mRNA and protein for AMH and its specific type II receptor (AMHRII) [38, 39]. AMH is thought to exert negative effects on cellular viability by binding to its receptor [38, 40]. Interestingly, a recent study in PCOS women showed that higher AMH level was associated with decreased endometrial thickness in ovulation induction cycles [41]. Thus, endometrial interaction with elevated serum AMH levels could explain the associated low implantation and live birth rates.

This study is limited by its retrospective design as well as the relatively small sample size. In addition, it is important to mention that ovarian stimulation in our study’s patients was performed with caution to minimize the risk of ovarian hyperstimulation syndrome (OHSS), as reflected by the relatively similar number of oocytes retrieved in the various AMH patient groups as well as similar rates of OHSS. The patients in this study were treated during a period preceding the currently established protocols of GnRH antagonists for pituitary suppression and GnRH agonist trigger for those at risk of OHSS, and it is interesting to speculate whether a more aggressive approach to ovarian stimulation with freeze-all strategy would have led to a higher live birth rate in the high AMH group. This is suggested by a recent study showing that frozen embryo transfer is associated with higher LBR than fresh transfer in PCOS women [42]. Interestingly, De Vos et al. reported that live birth outcomes in PCOS women with hyperandrogenic phenotypes, as compared to other PCOS phenotypes, remain lower also as cumulative outcomes after taking into account both fresh and frozen embryo transfers [19]. Further larger studies looking specifically at LBR outcomes according to AMH levels and PCOS phenotypes in frozen embryo transfers using contemporary freeze-all cycle protocols should help to clarify the relationship.

In conclusion, this study found for the first time that PCOS women with low serum AMH levels (< 3.32 ng/ml) had significantly higher live birth rates than those with average or high serum AMH levels following ART. There were several other ART outcomes that correlated inversely with AMH concentrations. While the reason may not be clear, it may be associated with the positive link between AMH, testosterone and PCOS severity. The results of this study underscore the potential utility of AMH and other phenotypic features of PCOS rather than the basic diagnostic criteria of the disorder in treatment individualization and counseling in daily ART practice. PCOS is a multifaceted, heterogenous disease that is difficult to characterize and additional studies are necessary to fully understand how AMH and ART outcomes are related in various PCOS phenotypes. Such new knowledge will contribute to developing better strategies to improve the likelihood of a live birth in this special patient group.

## Data Availability

The datasets used and/or analyzed during the current study are available from the corresponding author on request.

## Abbreviations

AMH: Antimullerian hormone
ART: Assisted reproductive technology
BMI: Body mass index
CPR: Clinical pregnancy rate
FSH: follicle stimulating hormone
hCG: human chorionic gonadotropin
LBR: live birth rate
LH: Luteinizing hormone
OHSS: Ovarian hyperstimulation syndrome
PCOS: polycystic ovarian syndrome
ROC: Receiver Operating Characteristic

## References

[1] La Marca A, Sighinolfi G, Radi D, Argento C, Baraldi E, Artenisio AC, Stabile G, Volpe A (2010). Anti-Mullerian hormone (AMH) as a predictive marker in assisted reproductive technology (ART). Hum Reprod Update.

[2] Tal R, Seifer DB (2017). Ovarian reserve testing: a user's guide. Am J Obstet Gynecol.

[3] Durlinger AL, Visser JA, Themmen AP (2002). Regulation of ovarian function: the role of anti-Mullerian hormone. Reproduction.

[4] Rotterdam EA-SPCWG: **Revised** 2003 Consensus on diagnostic criteria and long-term health risks related to polycystic ovary syndrome**.** Fertil Steril 2004, 81**:**19–25. 10.1016/j.fertnstert.2003.10.004 14711538

[5] Pellatt L, Hanna L, Brincat M, Galea R, Brain H, Whitehead S, Mason H (2007). Granulosa cell production of anti-Mullerian hormone is increased in polycystic ovaries. J Clin Endocrinol Metab.

[6] Dewailly D, Gronier H, Poncelet E, Robin G, Leroy M, Pigny P, Duhamel A, Catteau-Jonard S (2011). Diagnosis of polycystic ovary syndrome (PCOS): revisiting the threshold values of follicle count on ultrasound and of the serum AMH level for the definition of polycystic ovaries. Hum Reprod.

[7] Lin YH, Chiu WC, Wu CH, Tzeng CR, Hsu CS, Hsu MI (2011). Antimullerian hormone and polycystic ovary syndrome. Fertil Steril.

[8] Nardo LG, Yates AP, Roberts SA, Pemberton P, Laing I (2009). The relationships between AMH, androgens, insulin resistance and basal ovarian follicular status in non-obese subfertile women with and without polycystic ovary syndrome. Hum Reprod.

[9] Pigny P, Jonard S, Robert Y, Dewailly D (2006). Serum anti-Mullerian hormone as a surrogate for antral follicle count for definition of the polycystic ovary syndrome. J Clin Endocrinol Metab.

[10] Piouka A, Farmakiotis D, Katsikis I, Macut D, Gerou S, Panidis D (2009). Anti-Mullerian hormone levels reflect severity of PCOS but are negatively influenced by obesity: relationship with increased luteinizing hormone levels. Am J Physiol Endocrinol Metab.

[11] Tal R, Seifer DB, Khanimov M, Malter HE, Grazi RV, Leader B (2014). Characterization of women with elevated antimullerian hormone levels (AMH): correlation of AMH with polycystic ovarian syndrome phenotypes and assisted reproductive technology outcomes. Am J Obstet Gynecol.

[12] La Marca A, Orvieto R, Giulini S, Jasonni VM, Volpe A, De Leo V (2004). Mullerian-inhibiting substance in women with polycystic ovary syndrome: relationship with hormonal and metabolic characteristics. Fertil Steril.

[13] Brodin T, Hadziosmanovic N, Berglund L, Olovsson M, Holte J (2013). Antimullerian hormone levels are strongly associated with live-birth rates after assisted reproduction. J Clin Endocrinol Metab.

[14] Iliodromiti S, Kelsey TW, Wu O, Anderson RA, Nelson SM (2014). The predictive accuracy of anti-Mullerian hormone for live birth after assisted conception: a systematic review and meta-analysis of the literature. Hum Reprod Update.

[15] Tal R, Tal O, Seifer BJ, Seifer DB (2015). Antimullerian hormone as predictor of implantation and clinical pregnancy after assisted conception: a systematic review and meta-analysis. Fertil Steril.

[16] Kaya C, Pabuccu R, Satiroglu H (2010). Serum antimullerian hormone concentrations on day 3 of the in vitro fertilization stimulation cycle are predictive of the fertilization, implantation, and pregnancy in polycystic ovary syndrome patients undergoing assisted reproduction. Fertil Steril.

[17] Xi W, Gong F, Lu G (2012). Correlation of serum anti-Mullerian hormone concentrations on day 3 of the in vitro fertilization stimulation cycle with assisted reproduction outcome in polycystic ovary syndrome patients. J Assist Reprod Genet.

[18] Alebic MS, Stojanovic N, Duhamel A, Dewailly D (2015). The phenotypic diversity in per-follicle anti-Mullerian hormone production in polycystic ovary syndrome. Hum Reprod.

[19] De Vos M, Pareyn S, Drakopoulos P, Raimundo JM, Anckaert E, Santos-Ribeiro S, Polyzos NP, Tournaye H, Blockeel C (2018). Cumulative live birth rates after IVF in patients with polycystic ovaries: phenotype matters. Reprod BioMed Online.

[20] Ramezanali F, Ashrafi M, Hemat M, Arabipoor A, Jalali S, Moini A (2016). Assisted reproductive outcomes in women with different polycystic ovary syndrome phenotypes: the predictive value of anti-Mullerian hormone. Reprod BioMed Online.

[21] Seifer DB, Baker VL, Leader B (2011). Age-specific serum anti-Mullerian hormone values for 17,120 women presenting to fertility centers within the United States. Fertil Steril.

[22] Buyuk E, Seifer DB, Illions E, Grazi RV, Lieman H (2011). Elevated body mass index is associated with lower serum anti-mullerian hormone levels in infertile women with diminished ovarian reserve but not with normal ovarian reserve. Fertil Steril.

[23] Kelsey TW, Wright P, Nelson SM, Anderson RA, Wallace WH (2011). A validated model of serum anti-mullerian hormone from conception to menopause. PLoS One.

[24] Seifer DB, MacLaughlin DT, Christian BP, Feng B, Shelden RM (2002). Early follicular serum mullerian-inhibiting substance levels are associated with ovarian response during assisted reproductive technology cycles. Fertil Steril.

[25] Visser JA, de Jong FH, Laven JS, Themmen AP (2006). Anti-Mullerian hormone: a new marker for ovarian function. Reproduction.

[26] Pellatt L, Rice S, Mason HD (2010). Anti-Mullerian hormone and polycystic ovary syndrome: a mountain too high?. Reproduction.

[27] Garg D, Tal R (2016). The role of AMH in the pathophysiology of polycystic ovarian syndrome. Reprod BioMed Online.

[28] Jonard S, Dewailly D (2004). The follicular excess in polycystic ovaries, due to intra-ovarian hyperandrogenism, may be the main culprit for the follicular arrest. Hum Reprod Update.

[29] Pigny P, Merlen E, Robert Y, Cortet-Rudelli C, Decanter C, Jonard S, Dewailly D (2003). Elevated serum level of anti-mullerian hormone in patients with polycystic ovary syndrome: relationship to the ovarian follicle excess and to the follicular arrest. J Clin Endocrinol Metab.

[30] Grossman MP, Nakajima ST, Fallat ME, Siow Y (2008). Mullerian-inhibiting substance inhibits cytochrome P450 aromatase activity in human granulosa lutein cell culture. Fertil Steril.

[31] Pellatt L, Rice S, Dilaver N, Heshri A, Galea R, Brincat M, Brown K, Simpson ER, Mason HD (2011). Anti-Mullerian hormone reduces follicle sensitivity to follicle-stimulating hormone in human granulosa cells. Fertil Steril.

[32] Vendola KA, Zhou J, Adesanya OO, Weil SJ, Bondy CA (1998). Androgens stimulate early stages of follicular growth in the primate ovary. J Clin Invest.

[33] Weil S, Vendola K, Zhou J, Bondy CA (1999). Androgen and follicle-stimulating hormone interactions in primate ovarian follicle development. J Clin Endocrinol Metab.

[34] Qiao J, Feng HL (2011). Extra- and intra-ovarian factors in polycystic ovary syndrome: impact on oocyte maturation and embryo developmental competence. Hum Reprod Update.

[35] Filicori M, Cognigni GE, Ciampaglia W (2003). Effects of LH on oocyte yield and developmental competence. Hum Reprod.

[36] Rosas C, Orostica L, Poblete C, Carvajal R, Gabler F, Romero C, Lavandero S, Vega M (2016). Hyperandrogenism decreases GRP78 protein level and glucose uptake in human endometrial stromal cells. Reprod Sci.

[37] Tal R, Seifer DB, Arici A (2015). The emerging role of angiogenic factor dysregulation in the pathogenesis of polycystic ovarian syndrome. Semin Reprod Med.

[38] Wang J, Dicken C, Lustbader JW, Tortoriello DV (2009). Evidence for a Mullerian-inhibiting substance autocrine/paracrine system in adult human endometrium. Fertil Steril.

[39] Carrarelli P, Rocha AL, Belmonte G, Zupi E, Abrao MS, Arcuri F, Piomboni P, Petraglia F (2014). Increased expression of antimullerian hormone and its receptor in endometriosis. Fertil Steril.

[40] Signorile PG, Petraglia F, Baldi A (2014). Anti-mullerian hormone is expressed by endometriosis tissues and induces cell cycle arrest and apoptosis in endometriosis cells. J Exp Clin Cancer Res.

[41] Gaba A, Horath S, Hager M, Marculescu R, Ott J (2019). Basal anti Mullerian hormone levels and endometrial thickness at midcycle can predict the outcome after clomiphene citrate stimulation in anovulatory women with PCOS, a retrospective study. Arch Gynecol Obstet.

[42] Chen ZJ, Shi Y, Sun Y, Zhang B, Liang X, Cao Y, Yang J, Liu J, Wei D, Weng N (2016). Fresh versus frozen embryos for infertility in the polycystic ovary syndrome. N Engl J Med.

